# Selection and academic success of medical students in Hamburg, Germany

**DOI:** 10.1186/s12909-018-1443-4

**Published:** 2019-01-16

**Authors:** Hubertus Meyer, Stefan Zimmermann, Johanna Hissbach, Dietrich Klusmann, Wolfgang Hampe

**Affiliations:** Department of Biochemistry and Molecular Cell Biology, University Medical Center Hamburg-Eppendorf (UKE), Martinistr, 52, 20246 Hamburg, Germany

**Keywords:** Medical school selection, Predictive validity, Knowledge test, Self-selection, Reciprocal suppression

## Abstract

**Background:**

Student selection at Hamburg medical school is based on the combination of a natural science knowledge test (HAM-Nat) and pre-university educational attainment.

**Method:**

Of the 1565 medical students enrolled in Hamburg from 2012 to 2015 about half were admitted by an entrance test, half by quotas. First, we analysed sociodemographic determinants of entrance test performance. Then, we used regression analysis to describe the interplay of variables in the prediction of study outcome, the role of sociodemographic factors, and differences in the calibration of educational attainment specific to German federal states.

**Results:**

Better performance in the entrance test was associated with age over 21, male gender, German nationality, first language German and both parents holding an academic degree – effect sizes were small. No differences were found for the birthplaces of parents (a proxy for migration background). Study outcome differed considerably among admission paths: Students admitted by entrance test or the quota for excellent pre-university educational attainment performed markedly better during the first 3 terms than students admitted by the waiting list quota and the quota for foreign students. Gender differences in study outcome were slight with better performance by males. The relation of pre-university educational attainment to study outcome was moderated by the federal state in which secondary schooling took place. Methods for the equating of state-specific grades are explored. The predictive validity of the HAM-Nat after correction for range restriction was *r* = .31. The relatively low value of this coefficient may be attributed to 3 factors: 1. self-selection of applicants which leads to a validity-enhancing effect that is not revealed by the predictor-outcome correlation, 2. reduction of variance due to a high selection ratio, and 3. high test difficulty, exceeding the demands of the medical curriculum.

**Conclusion:**

The HAM-Nat achieves a small amount of incremental validity over pre-university educational attainment. This effect, obtained from correlational analysis, underestimates the validity of the test, because it does not reflect the role of self-selection and other validity enhancing features of the selection process.

## Background

### Aims of the study

This study investigates the predictive validity of the Hamburg Natural Science Test (HAM-Nat), an entrance test for medical school. In Germany, medical schools select applicants according to pre-university educational attainment and optional tests, such as the “Test für Medizinische Studiengänge” (TMS) [1], an aptitude test similar to the United Kingdom Clinical Aptitude Test (UKCAT) [2]. Both tests predominantly measure cognitive ability – only basic knowledge of scientific facts is required. In contrast, the University Medical Center Hamburg-Eppendorf (Universitätsklinikum Hamburg-Eppendorf, UKE) and two other German medical schools developed a test of scientific knowledge: the HAM-Nat [3], an 80-items test, designed to capture the scope of knowledge in physics, chemistry, and biology generally conveyed during secondary school. The difference between the UKCAT and the HAM-Nat is a difference between cognitive ability and positive knowledge of facts. “Cognitive ability”, “reasoning ability” or “intellectual aptitude” are largely synonymous labels for “intelligence” which has been shown to be the single most potent predictor of success in education and occupation [4, 5]. However, in the particular context of selection for medical school, cognitive ability seems to be of limited use as a predictor of academic success [6], mainly because the high level of pre-university educational attainment required for application assures that cognitive ability is a given for most students. Instead, variation in study success is more strongly determined by factors other than cognitive ability; mainly personality traits such as achievement motivation, conscientiousness, and emotional stability [7, 8]. A knowledge test reflects such factors because it demands persistent effort. Moreover, it is well equipped to predict success in written exams during the initial stages of the medical curriculum, as test items resemble exam items.

In a critique of UK medical student selection, Harris et al. (2015) [9] speak out against the use of tests which neither depend on knowledge, nor seem to have substantial predictive validity, specifically the UKCAT and Situational Judgement Tests (SJTs). Instead, they advocate the development of a standardised, nation-wide science knowledge test used in conjunction with pre-university educational attainment. This test would be similar to the HAM-Nat analysed in this article. Although differences between the properties of the HAM-Nat and the UKCAT cannot simply be attributed to the difference between knowledge and cognitive ability, we can at least assess whether the HAM-Nat surpasses the modest level of validity reported for the UKCAT, and how HAM-Nat scores relate to pre-university educational attainment and the sociodemographic factors analysed in the UKCAT-12 study [2]. In addition, we will compare the academic performance of students selected by HAM-Nat scores with students admitted by a quota instead of an entrance test. Finally, we will address a problem of fairness that arises from the calibration of educational attainment in Germany which corresponds to a similar problem in the UK with grades obtained from selective vs. non-selective schools.

### Pre-university educational attainment

Pre-university educational attainment (PEA) has consistently been found to be the single best predictor of higher education success [10]. In the UKCAT-12 study, PEA correlated with a global measure of academic success at medical schools by *r* = .36. Even though PEA is confounded with many factors such as differences in school curricula, assessment methods and use of scale, it still seems to reflect robustly what McManus has called “academic backbone” [11]: the capability to navigate the educational system successfully. This capability entails not only cognitive ability but also motivation, emotional stability and conscientiousness.

In the UK the predictability of academic success by PEA (represented by A-levels) depends on the type of school. The same A-level grade relates to higher performance at medical school when obtained from a non-selective school in contrast to a selective school. Thus, A-levels from non-selective schools are undervalued with respect to their capacity to predict medical school performance [2, 12]. In Germany, conditions are similar. The German secondary school-leaving certificate is termed Abitur. It is equivalent to A-levels and is likewise a precondition for university application. As the Abitur grade is standardised only within federal states, but not nation-wide, it reflects differences between the grading policies of federal states. Federal states also differ in their proportion of secondary school graduates. In Bavaria, for example, 31.6% of all school leavers achieve their Abitur, whereas in Hamburg the respective number is 57.7%. One might expect mean performance in secondary school to drop as a higher proportion of pupils is recruited; however, the means of the Abitur grade do not differ much between federal states. In the above example, it is possible that the pool of pupils willing and able to achieve secondary school graduation is smaller in Bavaria than in Hamburg for reasons specific to that federal state, e.g. a culture that places a lower emphasis on higher education, more attractive alternatives to secondary school, or higher requirements for entering secondary school. It is also conceivable that secondary schools in Hamburg assign grades more leniently than those in Bavaria [13]. These schools may have lowered their standards for pedagogic reasons, accommodating the influx of pupils who would not have been admitted to secondary school in more selective federal states. Studies using independent measures of scholastic ability show that in fact the same Abitur grade is related to different levels of competence depending on the federal state in which it was achieved [13]. At the University Medical Center Hamburg-Eppendorf (UKE) the Abitur grade determines applicant selection in two steps: 1. it serves as a threshold for admission to the entrance test, and 2. it is combined with the result of the entrance test to produce a rank order of eligibility. Thus, state specific differences in the calibration of the Abitur grade doubly influence the selection procedure. In this study we will assess this influence and briefly discuss methods to control it.

### Cognitive aptitude and knowledge

In 1928 the Medical College Admission Test (MCAT) was developed in the USA to complement PEA in the selection of undergraduate medical students. The correlation of MCAT scores with study success has been reported at *r* = .39 in a meta-analysis of studies from 1991 and 2006 [14]. After correction for range restriction, the estimated predictive validity is *r* = .43. The MCAT consists of four subtests: biological sciences, physical sciences, verbal reasoning and a writing sample. The subtest for biological sciences, requiring more knowledge than other subtests, yields high predictive validity.

The United Kingdom Clinical Aptitude Test (UKCAT) gives more weight to cognitive aptitude and less to knowledge than the MCAT. In the UKCAT-12 study, UKCAT scores correlated with medical school outcome by *r* = .15, and with PEA by *r* = .36 [2]. After taking PEA into account using regression analysis, the beta coefficient was = .06 which means that UKCAT scores yielded almost no incremental predictive power above PEA. However, these coefficients were based only on the subgroup of applicants who had been admitted to study medicine. They do not properly represent the predictive validity of the UKCAT, which would be the correlation in the total group of all applicants including those who were rejected. In a further article [6], correction for range restriction with a method described by Hunter et al. (2006) [15] was applied. The predictive validity of the UKCAT, conceived as the correlation of true scores for both predictor and outcome (construct-level predictive validity), was estimated to be *r* = .23.

### Gender

Female students are overrepresented at medical schools [16, 17]. A report from 2014 showed that 64.5% of medical students in Germany were female [18]. More females than males graduate from secondary school and females attain better grades than males, therefore their chances for admission to medical school are higher than those of males. Studies from other countries reveal a mixed picture of better performance at medical school in males [19], in females [2, 20], or equal success [17]. In a review, Ferguson [21] concluded that “women tend to perform better than men in their medical training and are more likely to attain honours. Women also tend to perform better in clinical assessments … However, these differences were small.”

### Socio-economic status

Socio-economic status seems to bear little or no relationship to medical school performance once educational attainment is taken into account [2]. In 2009, about 53% of all university graduates in Germany had at least one parent holding a university degree [22]. At medical schools this proportion was even higher, ranging from 68% to 72% [22, 23]. Only 5% of medical students had parents with no secondary education. About one quarter of medical students in Germany had at least one parent with a medical degree [18, 22], in other western countries this share is about 15% [24]. Having one “medically qualified” parent has been shown to be associated with successful application [25], less drop-out [26], and choice of a high-prestige, high-income medical specialty [27]. Effects are small and do not emerge consistently [28].

### Ethnicity

Nationality, ethnicity, and race are distinct but closely related features each based, to different degrees, on administrative acts, shared culture and shared genes, respectively. The heterogeneity of these categories within a nation’s population complicates international comparison. One feasible categorisation is the coarse distinction between white and non-white used in a meticulous meta-analysis of academic performance in the UK, based on 22 studies [29]. Students of non-white ethnicity underperformed compared to white students by 0.42 standard deviations (Cohen’s *d*). Similar results were reported in other studies from the UK [2, 30, 31].

## Method

### Study group

Medical schools in Germany are free to select 60% of their students through procedures of their own choice. The other 40% must be selected according to quotas: (1) excellent PEA, (2) waiting list, (3) students from foreign nations, and (4) miscellaneous groups, e.g. hardship, medical officers of the Federal Armed Forces. Entering the waiting list improves chances of admittance proportionately to the number of years elapsed since high-school graduation. For the discretionary part of the selection procedure, Hamburg applies an entrance test of scientific knowledge, the HAM-Nat [3, 32, 33]. Every year, about 115 applicants with the highest combined PEA and HAM-Nat scores are admitted directly. The 200 applicants following in rank order are invited to an additional test of social competence – a Multiple mini-interview called HAM-Int [34, 35] . The 100 applicants with the highest scores (a weighted combination of PEA, HAM-Nat, and HAM-Int) were admitted. Even though selection by social competence slightly modified selection by scientific knowledge, we will not consider the impact of the HAM-Int in this article to simplify analysis. From 2012 to 2015, a total of 9454 applicants was registered, 4615 were invited to the HAM-Nat, 3511 sat the test and 794 were admitted and enrolled (22.6% of tested applicants). Invitations to the HAM-Nat were extended to the top 1200 applicants based on PEA. Applicants from other EU-countries made up 3.1% of test takers and 2.7% of students enrolled through the HAM-Nat. Additionally, 771 students were accepted through the aforementioned quotas, yielding the total study group size of 1565 students.

### Measures

We distinguish three sets of measures: outcome, selection, and demographics. Almost all measures are plagued by a small number of missing values, therefore sample sizes may vary for different measures. If such variation is so minute that results are not affected, we will not show it. Italicised variable names refer to measures that enter statistical analyses while roman variable names refer to the corresponding concepts. The name for a dichotomous variable denotes the index category coded as 1, and ends with the number 1, e.g. *MaleGender1* means 1 if male, 0 if female. The name for a categorical variable with 4 categories ends with C4, variables without numerical endings are continuous, variables with the prefix z are standardised with mean = 0 and SD = 1.

### Selection

*AdmissionPathC5:* (1) entrance test (HAM-Nat), (2) quota excellent PEA, (3) quota waiting list, (4) quota foreign nation, (5) quota others/unknown.

*zEduAttain:* Pre-university educational attainment (PEA) derived from the German Abitur grade. The Abitur grade is an overall evaluation of performance in secondary school on a scale ranging from 1.0 as the highest score to 4.0 as the lowest. The range of applicants invited to the Hamburg admission test was 1.0–1.9. We used the negative of the Abitur grade to conform to the intuitive interpretation of scores wherein higher values equate to better performance. *zEduAttain* is the standardised negative Abitur grade and, on a conceptual level, is referred to as PEA score.

*zFederalBonus:* Federal states differ in educational policies, which effects the calibration of the Abitur grade. *zFederalBonus* is constructed to reflect the bonus provided by an inflationary assignment of good grades as contrasted to the malus provided by restrictive assignment. It is the standardised product of the proportion of pupils reaching the Abitur in a federal state with mean educational attainment in this state. Under the assumption of a uniform distribution of abilities across federal states, *zFederalBonus* reflects a bonus resulting from extensive recruitment to secondary school and/or lenient grading policies as contrasted to a malus from restrictive recruitment and/or strict grading policies.

*zHAMNat:* Standardised scores of the 80-item test of natural science knowledge that has been used for student selection in Hamburg since 2008. Of the 794 students admitted between 2012 and 2015 to Hamburg medical school by entrance test, 18.1% made two attempts and 2.4% more than two. The score of the last attempt is counted as the HAM-Nat-score of a student.

*MultipleAttempts1*: (1) HAM-Nat attempted in more than 1 year, (0) only one attempt.

### Outcome

The medical curriculum introduced to the UKE in 2012 is composed of three consecutive blocks of sub-curricula, P1, P2, and P3. Each block represents a different set of six learning modules that are assessed by written exams, oral and practical examinations. Block P1 spans the first three semesters and covers the “musculoskeletal system, heart, circulatory system, lung, molecules, genes, cells, ontogenetic development, and body functions”. Each of the six modules ends with a mandatory final examination in the form of a multiple choice-test, oral examination, test of practical skills, or a combination thereof. Our outcome measure is restricted to performance in block P1, because only these results were available for the entire sample.

*OutcomeGroupC4:* Outcome of the curriculum after the first three terms as documented in October 2016: (1) failed: passed none of the required modules (six modules in admission years 2012 to 2014, and four in 2015), (2) lagged: passed at least one, but not all required modules (3) resat: passed all required modules but resat at least one written exam, (4) no resitting: passed all required exams at first attempt.

*zOutcomeOverall*: Mean score of the performance ratings for the modules of block P1 attended thus far, regardless of whether a module was passed or not. If no module score had been obtained, missing was assigned. Distribution of the module performance rating is left-skewed, mainly because some students who severely lagged or dropped out had very low scores. To improve scale quality we used a normal score transformation (SPSS command Rank with Fraction = Blom). This transformation ranks the values of a variable from lowest to highest and matches these ranks to equivalent ranks generated from a standard normal distribution. This variable corresponds to “OverallMark” in the UKCAT-12 study [2].

### Demographic

Basic demographic information was obtained from the enrolment records for the study sample:

*AgeLow1:* Age (1) 21 or older at the time of admission, (0) under 21

*MaleGender1:* Gender (1) male, (0) female

*NationalityGerman1:* Nationality German (1) German, (0) not German

*NationalityC6:* Nationality of student (1) German, (2) Western European, (3) Eastern European, (4) Middle Eastern, (5) Asian, (6) Other

Additional demographic information shown in the following six variables was only available for the entrance test group. In this group 782 of 794 (98.5%) applicants answered an optional demographic questionnaire.

*FirstLanguageGerman1:* Is German the first language of the student? (1) yes, (0) no

*ApplicantParentsBornC4:* Applicant and parents were born in (1) Germany / Germany / Germany, (2) Germany / foreign country / Germany, (3) Germany / foreign country / foreign country, (4) foreign country / foreign country / foreign country.

*ParentsBothGermanBorn1:* Were both parents born in Germany? (1) yes, (0) no

*ParentsEducationC5:* Highest educational level of parents: (1) both academics, (2) one academic, (3) both Abitur, (4) one Abitur, (5) none Abitur

*ParentsBothAcademic1:* Do both parents hold an academic degree? (1) yes, (0) no

*ParentAcademic1:* Does at least one parent hold an academic degree? (1) yes, (0) no

*ParentIsMedicalProfess1:* Does at least one parent hold a medical degree? (1) yes, (0) no

### Correction for range restriction

The predictive validity of a test is defined as the correlation of its scores with an outcome criterion. However, the correlation between z*HAMNat* and *zOutcomeOverall* is only computable for those applicants who actually enrolled and produced outcome data (22.7%). We cannot naively take this correlation as an estimation of predictive validity because it only reflects outcome differentiation amongst successful applicants [36]. The first correction method we used is Thorndike’s case C formula for indirect selection [37] which estimates predictive validity as a function of the diminution of the standard deviation caused by selection, and the correlations between z*HAMNat*, *zEduAttain* and *zOutcomeOverall* (the influence of *zEduAttain* renders the selection by HAM-Nat indirect). The second method is a Bayesian type of estimation: Multiple Imputation by Chained Equations (= MICE, [38]).

## Results

### Entrance test and demographic factors

In the entrance test path, 3511 applicants sat the test (Table 1). 93.7% of applicants completed the optional demographic questionnaire. Incompletion and non-completion of the questionnaire correlated with low HAM-Nat scores. Applicants aged 21 and older performed better than younger ones (*d* = 0.23), and males performed better than females (*d* = 0.51). Applicants from Germany scored slightly higher than applicants from other EU-countries (*d* = 0.17). Scores were hardly affected by whether or not German was the first language (*d* = 0.11). German applicants whose parents were born in a foreign country (a proxy for migration background) performed equally well in the HAM-Nat to applicants whose parents were born in Germany. When both parents were academics, HAM-Nat scores were slightly higher than when only one parent was an academic (*d* = 0.10) or when no parent was an academic (*d* = 0.21). When a parent held an academic degree, it was irrelevant whether or not this degree was medical (*d* = 0.08). These results pertain to all applicants who sat the test. In the group of applicants who were finally admitted to the course, all differences in entrance test performance disappeared as a result of the range restriction imposed by selection.

**Table 1:** HAM-Nat results by demographic characteristics, all applicants who sat the HAM-Nat 2012–2015, *n* = 3511

	*n*	zHAMNat Mean	zHAMNat SD	*p*(*F*)	Cohen’s *d*
Age				<.001	
Under 21	2248	−0.084	0.972		ref
21 and older	1263	0.150	1.028		0.23
Gender				<.001	
Female	2296	−0.172	0.951		ref
Male	1215	0.326	1.006		0.51
Nation				.048	
Germany	3388	0.006	0.994		ref
Other nation	123	−0.175	1.120		−0.17
German is first language				<.001	
Yes	2760	0.066	0.987		ref
No	529	−0.043	0.974		−0.11
missing	222	−0.721	0.914		−0.83
Applicant/ parent1/ parent2 were born in				<.001	
Germany/ Germany/ Germany	2373	0.057	0.917		ref
Germany/ for. Country/ Germany	314	0.035	0.961		−0.02
Germany/ for. Country / for. Country	452	0.074	1.061		0.02
for. Country / for. Country / for. Country	96	−0.050	1.027		−0.11
missing, miscellaneous combinations	276	−0.649	1.362		−0.61
Parent’s education				<.001	
Both parents academic	1353	0.143	0.895		ref
One parent academic	926	0.048	0.958		−0.10
Both parents Abitur	194	−0.051	1.005		−0.20
One parent Abitur	243	−0.100	0.943		−0.26
No parent Abitur	535	−0.051	1.010		−0.20
missing	260	−0.678	1.011		−0.86
If academic: Is parent a medical doctor?				.069	
At least one parent a medical doctor	830	0.154	1.004		ref
At least one parent academic but not medical	1449	0.076	0.930		−0.08

### Entrance test and pre-university educational attainment as predictors of study outcome

**Fig. 1:**
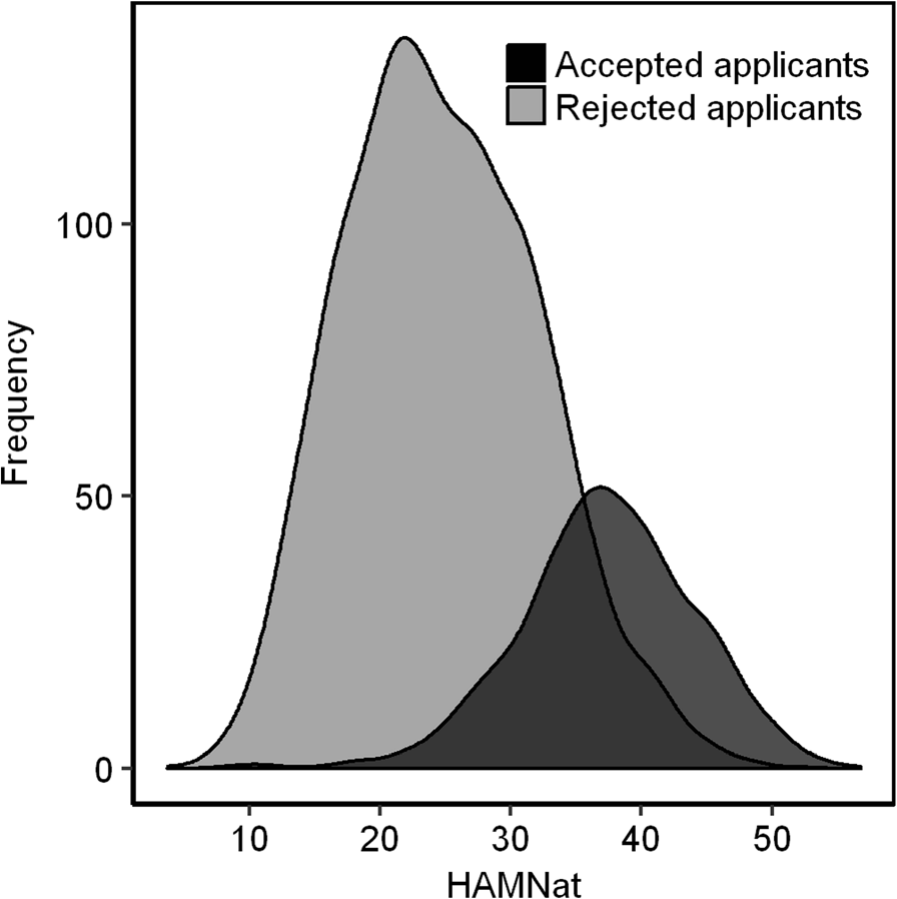
Distribution of HAM-Nat scores for admitted (dark grey) and rejected (light gray) applicants

HAM-Nat scores were distributed fairly normally (Fig. 1). The subgroup of admitted applicants was not separated from the total group by a clear-cut vertical line, as would be expected in a selection by one single criterion, but showed some blurring since educational attainment and social competence (HAM-Int) were also factored into admission decisions.

**Fig. 2:**
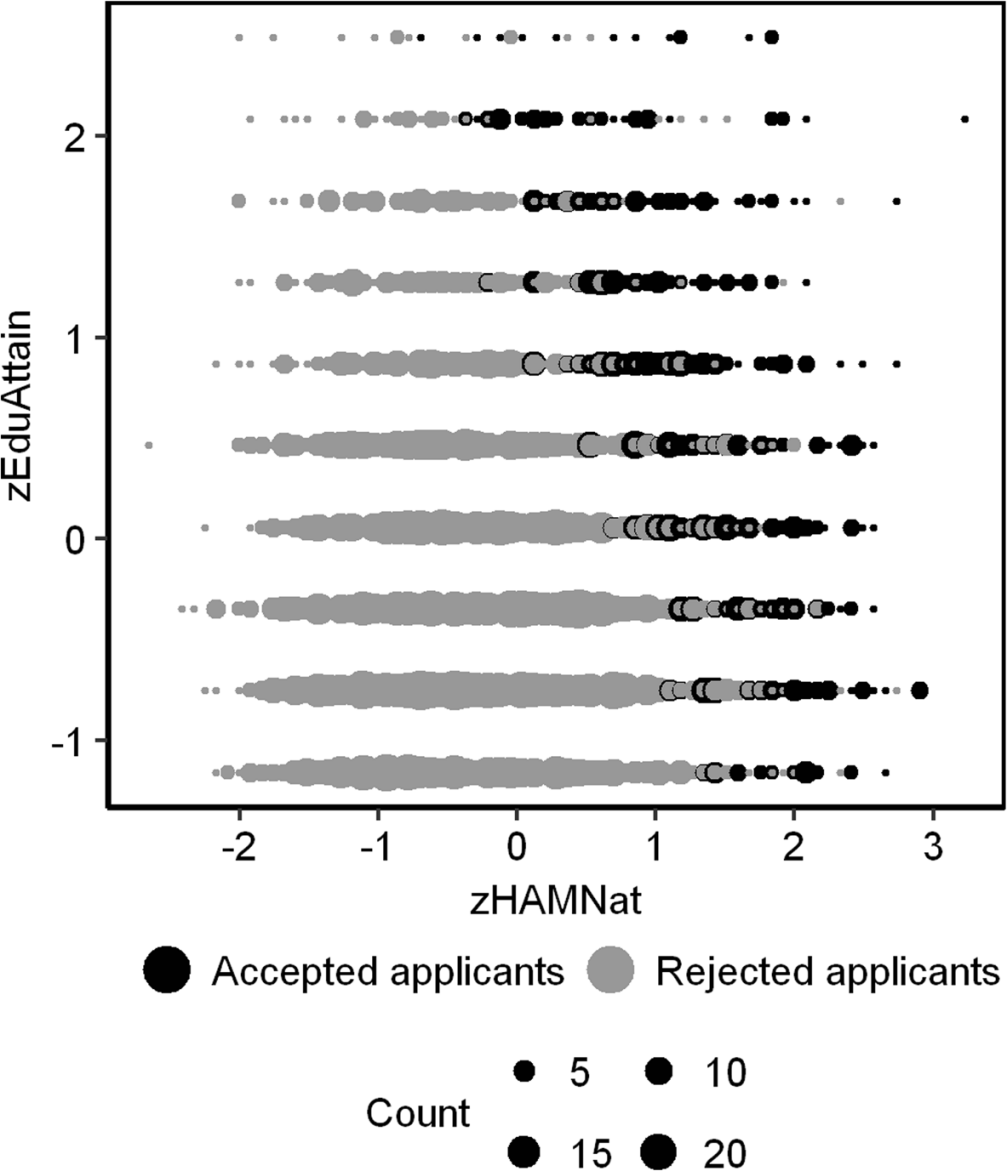
Scattergram of zHAMNat and zEduAttain

Within the total group of applicants who sat the test (*n* = 3511), the correlation between *zHAMNat* and *zEduAttain* was virtually zero (*r* = −.064). However, within the subgroup of admitted applicants (*n* = 794) it was markedly negative (*r* = −.522). This negative correlation reflects the admission procedure: High test performance compensated low PEA, and vice versa. Applicants who scored poorly on both were rejected, therefore the lower left area of the scattergram was excluded by the selection procedure while the upper right area was retained (Fig. 2).

In the group of students admitted by entrance test only, very few failed or lagged (Table 2). The four outcome groups (1) failed, (2) lagged, (3) resat at least one exam and (4) no resitting, differed neither in pre-university educational attainment, nor in their HAM-Nat scores.

**Table 2:** Study outcome groups^a^ by educational attainment and HAM-Nat, students enrolled by entrance test^b^

	Study outcome group				
	Failed	Lagged	Resat exam	Without resitting	*p*(*F*)
zEduAttain					.089
mean z-value	0.283	−0.001	−0.083	0.104	
SD	1.061	1.013	0.961	0.901	
Cohen’s *d*	0.18	−0.11	−0.20	ref	
sample size	13	25	182	567	
zHAMNat					.620
mean z-value	0.001	0.108	0.052	0.142	
SD	1.072	0.834	0.856	0.863	
Cohen’s *d*	−0.14	−0.04	−0.10	ref	
sample size	15	25	183	569	

^a^OutcomeGroupC4: Failed: did not pass any module; Lagged: passed at least one module, but not all required modules after 3 terms; Resat exam: passed all required modules, but resat at least one written exam; Without resitting: passed all required modules at first attempt

^b^Samples sizes differ slightly because 5 students had no PEA score

We used *zHAMNat* and *zEduAttain* to predict study success measured on a continuous scale as mean credit points *(zOutcomeOverall*). We are using the customary term prediction, even though we can only fit a model to data we already know. True prediction would be applying a prediction rule found in this study to data from another study. Keeping this in mind, we used multiple regression to estimate the independent contributions of *zHAMNat* and *zEduAttain* expressed by beta weights (Table 3). The beta weight of .242 for *zHAMNat* in model 2 means: If *zHAMNat* moves up one standard deviation, *zOutcomeOverall* is expected to move up 0.242 of a standard deviation, independent of the level of *zEduAttain*.

**Table 3:** Regression models for predicting study outcome (zOutcomeOverall): Effect of demographic factors, only students enrolled by entrance test^a^

	beta	sig.	*R*	*R* change	*p*(*F*) change
Model 1Only PEA			.009	.009	.010
zEduAttain	.093	<.001			
Model 2 HAM-Nat added to model 1					
zEduAttain	.217	<.001	.051	.042	<.001
zHAMNat	.242				
Model 3 Interaction added to model 2			.056	.014	.148
zEduAttain	.206	<.001			
zHAMNat	.251	<.001			
zHAMNat*zEduAttain	−.050	.163			
MultipleAttempts1	−.054	.149			
Model 4 Gender added to model 3			.059	.003	.331
zEduAttain	.211	<.001			
zHAMNat	.279	<.001			
zHAMNat*zEduAttain	−.060	.105			
MultipleAttempts1	−.051	.170			
MaleGender1	.043	.245			
MaleGender1*zHAMNat	−.052	.309			
Model 5 Demographic factors added to model 4			.084	.025	.003
zEduAttain	.232	<.001			
zHAMNat	.306	<.001		.	
zHAMNat*zEduAttain	−.061	.099			
MultipleAttempts1	−.031	.409			
MaleGender1	.042	.247			
MaleGender1*zHAMNat	−.065	.196			
AgeLow1	−.008	.835			
NationalityGerman1	.017	.644			
FirstLanguageGerman1	.092	.039			
ParentsBothGermanBorn1	.067	.124			
ParentsBothAcademic1	.024	.523			
ParentIsMedicalProfess1	.032	.392			
Model 6 Federal state added to model 3			.072	.016	.043
zEduAttain	.233	<.001			
zHAMNat	.249	<.001			
zHAMNat*zEduAttain	−.058	.105			
MultipleAttempts1	−.046	.209			
Abitur grade from Schleswig-Holstein^b^	.071	.047			
Abitur grade from Lower Saxony^b^	.099	.008		.	
Abitur grade from North Rhine-Westphalia^b^	.024	.518			
Abitur grade from Baden-Württemberg^b^	.043	.238			

^a^Sample size for all models is *n* = 761 instead of *n* = 794 due to missing values in *zOutcomeOverall*, *zEduAttain*, and variables of the sociodemographic questionnaire

^b^Reference state is Hamburg

**Fig. 3:**
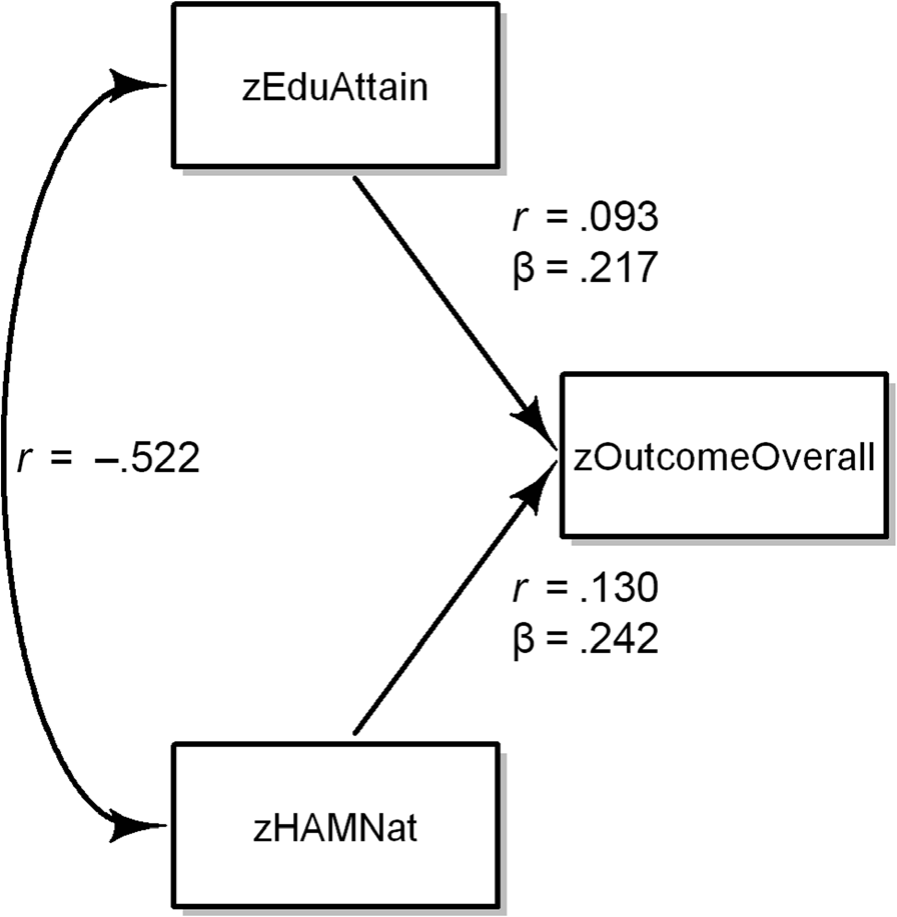
Reciprocal suppression in the prediction of zOutcomeOverall by zHAMNat and zEduAttain

If *zEduAttain* is the only predictor of *zOutcomeOverall,* its first order correlation, which in this case is equivalent to its beta weight, is *r* = beta = .093 (model 1 in Table 3). Similarly, the first order correlation between *zHAMNat* and *zOutcomeOverall* is *r* = beta = .130. If both variables are included in a multiple regression model (Model 2 in Table 3), a peculiar phenomenon occurs: the beta weights for *zHAMNat* and *zEduAttain* increase from .130 to .242 and .093 to .217, respectively. If a beta weight increases after the introduction of a new variable into a regression equation, this points to a suppressor effect (Fig. 3). A suppressor variable improves predictability by purging irrelevant variance from other predictor variables. The configuration at hand is called reciprocal suppression and has been thoroughly analysed [39]. Reciprocal suppression occurs whenever two predictors correlate negatively with each other (in this case *r* = −.522), and positively with a third variable. In such a configuration the predictors impede each other’s predictive power because whenever one predictor is high, the other tends to be low. The origin of the negative correlation between HAM-Nat and educational attainment is clear: It stems from the compensatory selection rule that excluded applicants with low scores in both variables.

The variables *zEduAttain* and *zHAMNat* provided significant incremental prediction of *zOutcomeOverall* above the contribution of the respective other variable. Model 3 refines prediction further. We know from an earlier study that interaction between *zHAMNat* and *zEduAttain* may occur [32]. Therefore, we included an interaction term in the regression equation. It failed the conventional significance level, and therefore only hints at a slight tendency for the HAM-Nat to more successfully predict outcome in applicants whose educational attainment is low rather than high. If applicants made multiple attempts at the HAM-Nat, this did not significantly lower prediction of *zOutcomeOverall*. Regression model 4 additionally includes gender and its interaction with HAM-Nat scores, both factors failing significance. However, a simple t-test of mean differences in outcome between males and females yielded a significant difference in favour of males corresponding to an effect size of *d* = 0.19.

Model 5 adds six demographic variables from the optional questionnaire answered by 98.5% of the test takers who were admitted. Study outcome did not depend on age or nationality of the student. It was also independent of whether or not parents were academics, held a medical degree, or were born in Germany (a proxy for migration background). Only for *FirstLanguageGerman1* a weak effect pointed toward lower study outcome among students who learned German as a second language.

**Fig. 4:**
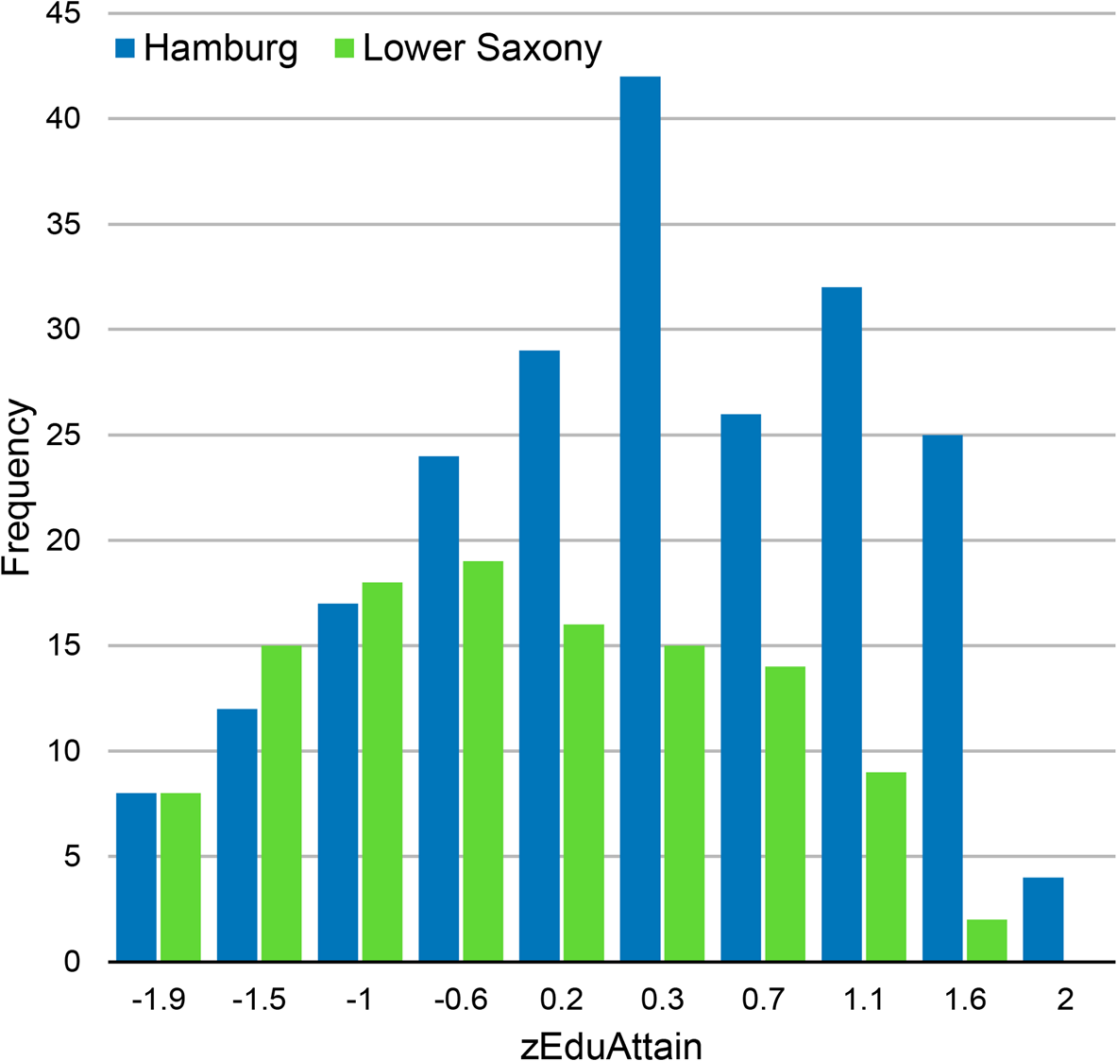
Distributions of zEduAttain in students from the federal states of Hamburg and Lower Saxony

**Fig. 5:**
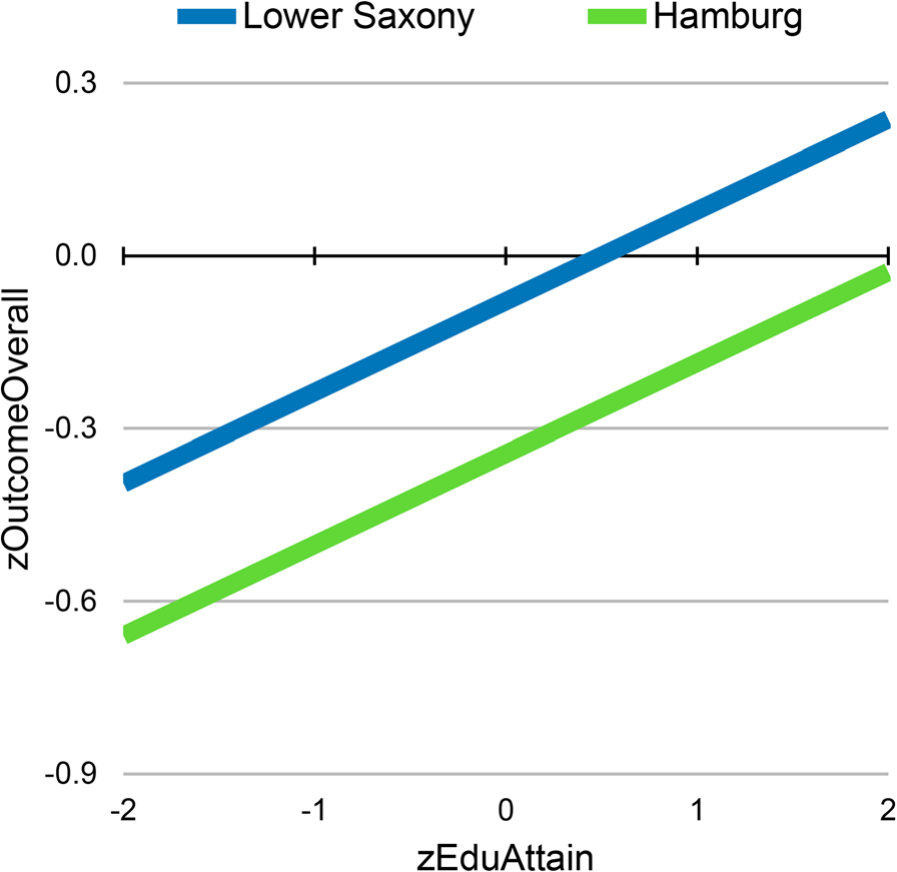
zEduAttain predicting study success: regression lines for Hamburg and Lower Saxony

In the entrance test group, the relation of PEA to study outcome differed among federal states. Depending on the federal state in which PEA-grade was obtained, the same grade corresponded to different levels of study outcome. As a consequence, federal state contributed significantly to the prediction of study outcome when added as a set of dummy variables into a regression equation in addition to *zEduAttain*, *zHAM-Nat*, the interaction of these variables, and a term reflecting multiple attempts (Table 3, Model 6). The largest difference occurred between Hamburg and Lower Saxony. If grades were obtained in Lower Saxony as opposed to Hamburg (Figs. 4 and 5), an increase of 0.265 standard deviations for study outcome was expected at all levels of PEA. These effects may be attributed to any factor associated with differences between federal states, e.g. quality of secondary schooling, or state-specific propensity to apply in Hamburg (self-selection). However, one potential factor looms large: different educational policies of the states. To capture such differences, we devised a measure of the relative bonus or malus associated with a federal state. *zFederalBonus* reflects leniency (high) vs. strictness (low) in the grading policy of a state. After *zFederalBonus* was entered into the regression equation, the effect for federal states, as shown in Table 3, model 6, disappeared. This might be taken to support the assumption that differences between states are largely differences of leniency vs. strictness in grading as reflected by the *zFederalBonus* variable*.*

### Correction for indirect range restriction

Range restriction due to selection reduces the standard deviation of the HAM-Nat by u = SD_selected_/SD_total_ = 0.75 and the first order correlation within the selected group to *r* = .130. Correction for indirect range restriction yielded a validity coefficient of *r* = .313 using Thorndike’s case C formula. The Bayesian method MICE yielded *r* = .304.

### Admission path, study outcome, and demographic factors as predictors of study outcome

**Fig. 6:**
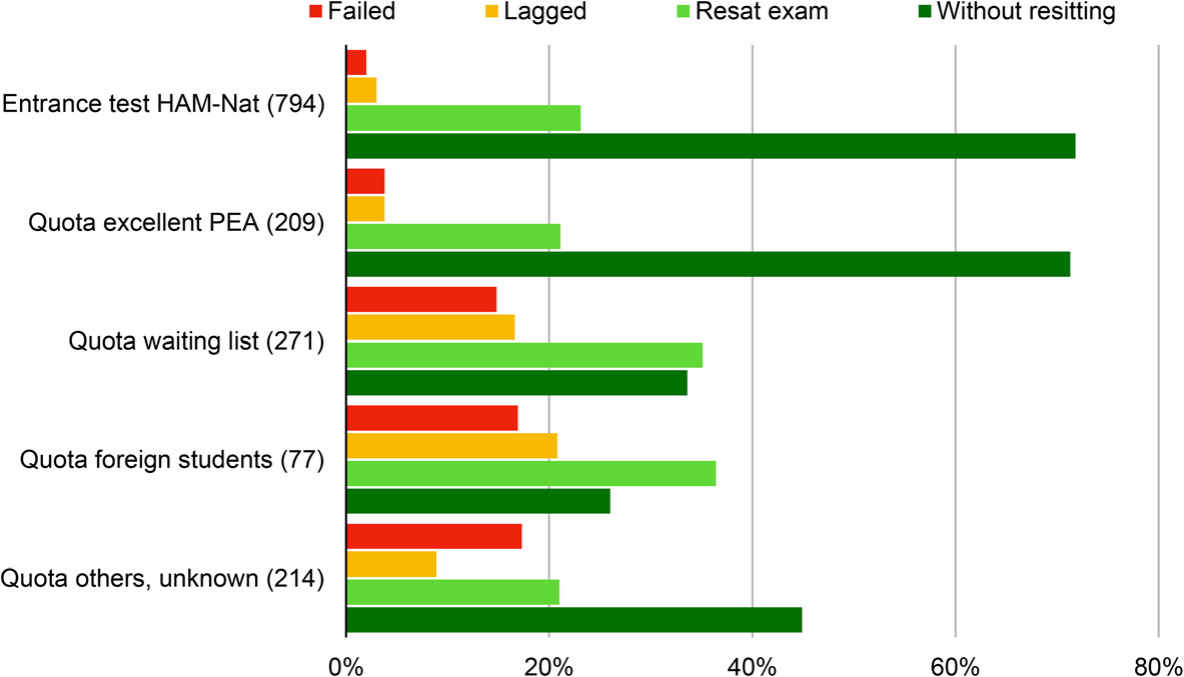
Study outcome by admission path, proportions add up to 100% within admission path group

Study outcome after the first three terms differed considerably among admission paths. Applicants admitted by entrance test and by quota for excellent PEA achieved the best outcome (Fig. 6), only about 5% of this group failed or lagged, and nearly three quarters completed the first three terms without resitting a single exam. Students from the waiting list and the foreign student quota group performed worse, about one third failed or lagged and only about one third succeeded without resitting.

The continuous measure *zOutcomeOverall* (mean of all credit points acquired in the first three terms) correspondeds to this pattern (Table 4) with the entrance test group and the excellence group achieving the highest scores, and foreign students and students from the waiting list the lowest. Differences in age are attributable to the fact that in the low performing waiting list group mean age is higher than in other groups. Male students achieved slightly higher *zOutcomeOverall* scores than females. Marked differences occurred with nationality: Students from Germany and Western Europe achieved higher scores than students from Eastern Europe, the Middle East and Asia.

**Table 4:** Study outcome^a^ by demographic characteristics, all students enrolled^b^

	*n*	zOutcome Mean	zOutcome SD	*p*(*F*)	Cohen’s *d*
Age				<.001	
Under 21	748	0.284	0.891		ref
21 and older	765	−0.277	1.020		−0.59
Gender				.006	
Female	878	−0.060	0.995		ref
Male	635	0.083	0.999		0.14
Admission path^c^				<.001	
HAM-Nat	786	0.268	1.083		ref
Quota excellence	208	0.477	0.824		0.22
Quota waiting list	256	− 0.675	0.901		−0.95
Quota foreign students	71	−0.994	0.687		−1.39
Quota others, unknown	192	−0.347	0.967		−0.60
Nation				<.001	
Germany	1391	0.055	0.985		ref
Western European	18	0.132	0.768		0.09
Eastern European	30	−0.648	0.922		−0.74
Middle East	37	−1.047	0.772		−1.27
Asia	23	−0.855	0.822		−1.00
Other nations	14	−0.021	1.077		−0.07

^a^zOutcomeOverall

^b^of the *n* = 1565 admitted students *n* = 52 did not attended at least one study module; in these cases zOutcomeOverall could not be computed

^c^HAM-Nat: Entrance test in combination with PEA

Quota excellence: Highest level of the Abitur grade. If there are more candidates than places, waiting time, social engagement and other criteria are considered; finally, a lottery decides

Quota waiting list: Admittance depends on the number of semesters that an applicant has waited. While on the waiting list, applicants are not expected to enrol at a German university

Quota foreign students: The federal state of Hamburg allows selection of about 5% of students from foreign countries outside the EU

Quota others, unknown: 35 students who studied medicine as an adjunct to other studies; 31 were medical officers of the Federal armed Forces; 3 cases of hardship; 46 students were admitted prior to 2012 through the quota for excellent pre-university educational attainment, the waiting list quota or the HAM-Nat, yet commenced their studies only after 2012 and 2015 due to interruption by military deployment; and 77 students whose path to admission could not be retrieved from the database of the university. An unknown part of this latter group is comprised of students who successfully sued for their admittance and students already enrolled in the study programme who swapped places with students enrolled at a different university

## Discussion

The HAM-Nat yielded incremental validity over pre-university educational attainment but the effect size was low. After correcting for indirect range restriction estimated predictive validity was *r* = .31. Membership in outcome groups (failed, lagged, resat exam, without resitting) was not related to HAM-Nat-scores. In terms of predictor-outcome correlation, the HAM-Nat performed slightly better than the UKCAT [2], but the bottom line is that neither of these two entrance tests provides much predictive information for later performance in the medical curriculum. In the final part of this discussion we will reflect on possible reasons, and argue why this finding cannot simply be taken as indicative of low validity. But first let us compare results with the UKCAT-12 study.

The German distinction between admission paths, particularly entrance test vs. quotas (Table 4), has no counterpart in the UK. Therefore, when considering the entrance test, we compare a highly selected group in Hamburg with a less selected group in the UK. A second difference pertains to the use of pre-university educational attainment (PEA). In the UK, PEA is used only once by defining the level required for entering the selection process, whereas in Hamburg PEA (Abitur grade) is used twice: first for shortlisting and second for ranking when combined with test results.

### Entrance test and pre-university educational attainment

The markedly negative correlation between HAM-Nat and PEA suppresses the relation of both variables with outcome measures, because the selection procedure removes all applicants with both low PEA and a low HAM-Nat-score. Controlling for PEA in a regression of the HAM-Nat on study outcome augments the correlation due to the alleviation of reciprocal suppression. In the UK, where no suppressor effect exists, including PEA into the regression strips the UKCAT of virtually all its predictive power due to the elimination of shared predictive variance.

### Entrance test results and demographic factors

Results of HAM-Nat and UKCAT are similar for gender, age, and nationality. In both studies, males achieve higher scores in the selection test than females, and older applicants (age 21 plus) perform better than younger ones. Nationality made a difference in the UKCAT-12 study (in favour of UK-nationals), but not in the entrance test group of the Hamburg-study, which contains only a few non-Germans from EU-countries. We included variables indicating birthplace of students’ parents (a proxy for migration background), and students’ native language. Only native language made a difference: Students for whom German was a foreign language performed slightly worse in the HAM-Nat than native German speakers. In the UKCAT-12 the entrance test was more predictive for females than for males, in the Hamburg-Study only a weak tendency in the same direction was observed.

### Study outcome and demographic factors in the total student body

This comparison applies to the total group of all medical students in both studies. In Hamburg, males performed slightly better than females in overall outcome, while in the UKCAT-12 study these differences were reverse. In both studies, younger students performed better than older students and this difference was more pronounced in Germany than in the UK due to the German waiting list quota.

The UKCAT-12 study yielded a large effect size in favour of white students. The Hamburg-Study provides no counterpart to the white/non-white distinction. What comes closest is the distinction between foreign and German nationality. A small proportion of students admitted via entrance test came from other EU countries (24/794). Their study success was equal to that of German students admitted in the same quota. In contrast, foreign students from non-EU countries who were admitted by a special quota were markedly less successful than German students from the entrance test group and the group with excellent PEA. However, these foreign students fare similar to German students admitted from the waiting list or from other particular paths to admission – all having in common a low level of selection requirements. Therefore, selection requirements need to be controlled for before trying to attribute the low performance of foreign students to more specific causes.

In the UK, it has been found that of two students with the same A-level grades, the student from a selective school will achieve lower overall marks in medical school than the student from a non-selective school [2]. A similar situation exists in Germany for federal states. Differences in state-specific educational policy assign an unearned bonus to some applicants and a malus to others. The present situation obviously disadvantages applicants from federal states with a strict grading policy such as Lower Saxony. We explored two methods to correct differences in the calibration of pre-university educational attainment (PEA). Both methods, percentiles and the stipulation of a constant relation to outcome, yielded large correction factors. A more general and systematic exploration of correction methods from 1980 arrives at the same conclusion [40]. Due to a recent decision by the German Constitutional Court, a correction for PEA from different states must be integrated in new selection laws by 2020.

### Selection effects

The correlation coefficient depends not only on causal relations between two or more variables, but also on the composition of the sample. An example for this is the strong negative correlation between HAM-Nat and educational attainment artificially produced by the selection procedure which excludes applicants low on both measures. In this case, the selection effect is transparent. A less transparent kind of selection occurs earlier: self-selection into the pool of applicants for medical school. Of all adolescents considering to study medicine, only a proportion finally decides to apply. After having reviewed the requirements, some will estimate their chances of success high enough to give it a try, and others will postpone application or give it up altogether. This process of self-selection seems to be largely hidden from the light of educational research. In Hamburg, extensive information about the HAM-Nat is provided on the medical school’s website. The use of the HAM-Nat for selection attracts applicants who judge themselves as highly competent in the natural sciences. Potential applicants know that a low level of educational attainment needs to be compensated by an excellent HAM-Nat, whereas a high level only requires a fairly decent HAM-Nat result. If all applicants anticipated their test results with reasonable accuracy and if they spent as much effort for test preparation as necessary to compensate their level of educational attainment, a negative relation between PEA score and HAM-Nat would be the consequence, and it would already exist on the day of the test – entirely produced by self-selection. However, studies in social psychology show that self-perception of competence is not tightly tethered to actual performance [41]. How accurately applicants assess their own competence is an open question.

A high PEA score usually indicates a good grasp of natural science, because school performance in mathematics, physics, chemistry, and biology enters into it. On this account, the relation between PEA score and HAM-Nat should be positive. However, as explicated above, the selection process probably counteracts this relation: A high PEA score does not need to be compensated by high competence in natural science, but a low PEA score does. Compensation would render the relation between PEA score and HAM-Nat negative. The zero-correlation we found between HAM-Nat and PEA score in the sample of all test takers probably reflects a balance between these two opposing influences. It seems to be largely caused by the deliberate selection process which rewards the effort of compensating relatively low grades.

### Why is predictive validity so low?

The correlation between entrance test and study success in this study is disappointingly low, even after correction for the effects of reciprocal suppression and range restriction. This finding is in line with results from similar studies [9], but why is it so? Three factors seem to be important:

1. *self-selection* into the pool of applicants based on self-appraisal and expectations about the test,
2. *reduction of variance* in predictor and outcome measures due to a high selection ratio,
3. *overshooting test difficulty* driven by the need to ever more differentiate.

#### Self-selection

Applicants in the Netherlands who decided to participate in a test procedure instead of a lottery had a lower drop-out rate [28, 42] and performed better in their studies [43]. Self-selection into test-participation as opposed to participation in a lottery predicted as much of the variance of drop-out as did the magnitude of actual test scores. Thus the predictive validity based on the correlation of test scores with the dichotomous drop-out variable reflected only half of the predictive power of the test – the other half was purely an effect of the decision to choose the test over the lottery. This validity-enhancing effect of self-selection is undetectable by methods derived from the predictor-outcome correlation.

Applicants are not just passive subjects of selection procedures, but actors who assess their resources, their motivation, and their chances [44]. Their reaction to the existence of a test should be counted as part of the selective power of a test, instigated through the perception of the test as a hurdle. A rational applicant would only prepare for the test if she judged her chances to be reasonably high. If competition is strong, preparation for application grows into an all-or-nothing decision. Then, the existence of the test has exerted its selective effect long before the first multiple choice box is checked. Self-selection is usually not appreciated as part of the validity of a test, however it would not occur without the test conspicuously existing and potential applicants responding to its existence. By this logic, a test may well promote an institution’s selection goals but fail to show this merit with the conventional correlative methods of validity assessment. Consider the following thought experiment: If on the day of the test, to the surprise of the assembled applicants, a lottery was offered and a random sample of 22.7% admitted, then the mere psychological effect of having expected the test with its consequence of self-selection into the group of applicants would influence acceptance, not actual test performance. With the currently estimated predictive validity corrected for range restriction of *r* = .31, a group randomly admitted from all test takers would not fare much worse than a group selected by test scores. Of course, such a game can only be played once. In order to instigate self-selection, the test needs to be conspicuously in place – in a similar sense as the consistent display of a good hand is required to efficiently bluff in a card game.

#### Reduction of variance

The variance of HAM-Nat scores in the test participants who are finally admitted (22.7%) is reduced as compared to the total group of all applicants. The limiting case of variance reduction would be a situation in which scientific knowledge in the accepted group is uniformly high. In this case, any study outcome that depends fully on scientific knowledge would also be expected to be uniformly high with no variance left to produce a correlation. With a variance reduction of 25% in the accepted group, we are not close to this limiting case, partly because other factors, PEA and an additional test of social competence also influence admission and thus render the relation of the HAM-Nat to outcome indirect. The corrections we applied for indirect range restriction have led us to estimate the predictive validity of the HAM-Nat at *r* = .31. Considering textbooks of psychometrics, this would normally not suffice to speak of substantive predictive information, but considering the accessory effect of self-selection induced by the HAM-Nat as a further contribution of unknown magnitude, an estimated predictive validity of *r* = .31 based on test scores may be judged sufficient, and it may be the most that is possible considering the circumstances. The high level of ability created by severe test selection also reduces outcome variance. In the end, virtually all applicants will easily master the natural science demands of the curriculum and the very success of the test will have erased the possibility to demonstrate its predictive validity – at least by using the variance left in accepted applicants. This holds, even when in fact the test works effectively through self-selection and in comparison to other procedures such as quotas.

#### Overshooting test difficulty

The difficulty of test items can be increased in order to improve differentiation in the decision zone around the 75% percentile, and in fact item difficulty did increase in the first years after the HAM-Nat was introduced. However, the level of knowledge in physics, chemistry, and biology recently required during the first three terms of the medical curriculum stayed on the same level or rose at a slower pace. The demands of the curriculum seem to be lower than the demands that need to be met for achieving a rank in the upper quartile of the HAM-Nat. To the extent that this is true, the relation of HAM-Nat-scores to study outcome is weakened because it differentiates in a region of ability that is above the region relevant for study success. This conjecture is in need of substantiation by research.

## Conclusion

The HAM-Nat added predictive information for study success above what was obtainable from PEA. Students selected by the HAM-Nat achieved study results equal to the group admitted due to excellent grades in secondary school and markedly better than students admitted by the waiting list quota and the quota for foreign students. As the test covers much of the medical curriculum of the first years, one might say that part of the natural science groundwork for medicine has been moved to the pre-university phase, by making its acquisition a precondition for admission. In preparing for the test, applicants experience the demands of the first terms of the medical curriculum, and thus can base their decision about whether or not to apply on realistic information. Recently, the test may have overshot its goal of assuring an adequate level of scientific knowledge. Yet, as we have seen, it has other functions as well: it signals a hurdle at the entrance to medical school and makes application a costly investment that requires serious motivation.

The knowledge test also lends legitimacy to the selection procedure. In our culture, selection procedures have to be impartial and fair, they should respond to no other features of applicants than merit. A test of scientific knowledge can easily be understood as a test of merit, because everyone with a suitable level of pre-university educational attainment has a chance to prepare for it by means virtually open to everyone. Those who put effort into this task should be rewarded. This works well as long as the number of applicants whose merits would justify acceptance does not largely exceed the number of vacant places. Such a discrepancy puts a strain on the selection procedure, because even when the marginal utility of further differentiation in terms of predictive validity drops, the need to differentiate does not subside. Such a development does not escape the attention of applicants and other educational stakeholders and therefore poses a problem of fairness and legitimacy that awaits to be solved.

## Abbreviations

Abitur grade (*Abiturnote*): Secondary school leaving grades, the negative of the Abitur grade corresponds to PEA
Abitur: Secondary school graduation
HAM-Int: Hamburg multiple mini-interview
HAM-Nat: Hamburg natural science test
MCAT: ‘Medical College Admission Test
MICE: Multiple Imputation by Chained Equations
PEA: Pre-university educational attainment
SES: Socio-economic status
SJT: Situational Judgement Test
TMS: Test für medizinische Studiengänge
UKCAT: United Kingdom Clinical Aptitude Test
UKE: University Medical Center Hamburg-Eppendorf

## References

[1] Trost G, Blum F, Fay E, Klieme E, Maichle U, Meyer M, et al. Evaluation des Tests für medizinische Studiengänge (TMS). Bonn 1998.

[2] McManus IC, Dewberry C, Nicholson S, Dowell JS (2013). The UKCAT-12 study: educational attainment, aptitude test performance, demographic and socio-economic contextual factors as predictors of first year outcome in a cross-sectional collaborative study of 12 UK medical schools. BMC Med.

[3] Hissbach J, Klusmann D, Hampe W (2011). Dimensionality and predictive validity of the HAM-Nat, a test of natural sciences for medical school admission. BMC Med Educ..

[4] Gottfredson LS (2002). g: Highly general and highly practical. The general factor of intelligence: how general is it?.

[5] Deary IJ, Strand S, Smith P, Fernandes C (2007). Intelligence and educational achievement. Intelligence.

[6] McManus IC, Dewberry C, Nicholson S, Dowell JS, Woolf K, Potts HWW (2013). Construct-level predictive validity of educational attainment and intellectual aptitude tests in medical student selection: meta-regression of six UK longitudinal studies. BMC Med.

[7] Higgins DM, Peterson JB, Pihl RO, Lee AG (2007). Prefrontal cognitive ability, intelligence, big five personality, and the prediction of advanced academic and workplace performance. J Pers Soc Psychol.

[8] Duckworth AL, Seligman MEP (2005). Self-discipline outdoes IQ in predicting academic performance of adolescents. Psychol Sci.

[9] Harris BH, Walsh JL, Lammy S (2015). UK medical selection: lottery or meritocracy?. Clinical Medicine.

[10] Trapmann S, Hell B, Weigand S, Schuler H (2007). Die Validität von Schulnoten zur Vorhersage des Studienerfolgs - eine Metaanalyse. Zeitschrift für Pädagogische Psychologie.

[11] McManus IC, Woolf K, Dacre J, Paice E, Dewberry C (2013). The academic backbone: longitudinal continuities in educational achievement from secondary school and medical school to MRCP(UK) and the specialist register in UK medical students and doctors. BMC Med.

[12] Mwandigha LM, Tiffin PA, Paton LW, Kasim AS, Böhnke JR (2018). What is the effect of secondary (high) schooling on subsequent medical school performance? A national, UK-based, cohort study. BMJ Open.

[13] Neumann M, Nagy G, Trautwein U, Lüdtke O (2009). Vergleichbarkeit von Abiturleistungen. Z Erzieh.

[14] Donnon T, Paolucci EO, Violato C (2007). The predictive validity of the MCAT for medical school performance and medical board licensing examinations: a meta-analysis of the published research. Acad Med.

[15] Hunter JE, Schmidt FL, Le H (2006). Implications of direct and indirect range restriction for meta-analysis methods and findings. J Appl Psychol.

[16] Larkins S, Michielsen K, Iputo J, Elsanousi S, Mammen M, Graves L (2015). Impact of selection strategies on representation of underserved populations and intention to practise: international findings. Med Educ.

[17] Griffin B, Hu W (2015). The interaction of socio-economic status and gender in widening participation in medicine. Med Educ.

[18] Jacob R (2015). Berufsmonitoring Medizinstudenten 2014: Ergebnisse einer bundesweiten Befragung.

[19] Riese A, Rappaport L, Alverson B, Park S, Rockney RM (2017). Clinical performance evaluations of third-year medical students and association with student and evaluator gender. Acad Med.

[20] Haidinger G, Frischenschlager O, Mitterauer L (2006). Reliability of predictors of study success in medicine. Wien Med Wochenschr.

[21] Ferguson E (2002). Factors associated with success in medical school: systematic review of the literature. BMJ.

[22] Schwarzer A, Gregor F (2012). Medizinerreport 2012 – Berufsstart und Berufsverlauf von Humanmedizinerinnen und Humanmedizinern. HIS Hochschul-Informations-System GmbH.

[23] Middendorff E, Apolinarski B, Poskowsky J, Kandulla M, Netz N (2013). Die wirtschaftliche und soziale Lage der Studierenden in Deutschland 2012. Sozialerhebung des Deutschen Studentenwerks, durchgeführt durch das HIS-Institut für Hochschulforschung Berlin.

[24] Voracek M, Tran US, Fischer-Kern M, Formann AK, Springer-Kremser M (2010). Like father, like son? Familial aggregation of physicians among medical and psychology students. High Educ.

[25] McManus IC, Richards P (1984). Audit of admission to medical school: I: acceptances and rejects. BMJ (Clin Res Ed)..

[26] Arulampalam W, Naylor R, Smith J (2004). Factors affecting the probability of first year medical student dropout in the UK: a logistic analysis for the intake cohorts of 1980–92. Med Educ.

[27] Vaglum P, Wiers-Fenssen F, Ekeberg Ø (1999). Motivation for medical school: the relationship to gender and specialty preferences in a nationwide sample. Med Educ.

[28] O'Neill L, Hartvigsen J, Wallstedt B, Korsholm L, Eika B (2011). Medical school dropout - testing at admission versus selection by highest grades as predictors. Med Educ.

[29] Woolf K, Potts HWW, McManus IC (2011). Ethnicity and academic performance in UK trained doctors and medical students: systematic review and meta-analysis. BMJ (Clin Res Ed).

[30] James D, Chilvers C (2001). Academic and non-academic predictors of success on the Nottingham undergraduate medical course 1970-1995. Med Educ.

[31] Yates J, James D (2006). Predicting the “strugglers”: a case-control study of students at Nottingham University medical school. BMJ (Clin Res Ed).

[32] Hissbach J, Feddersen L, Sehner S, Hampe W (2012). Suitability of the HAM-Nat test and TMS module “basic medical-scientific understanding” for medical school selection. GMS Z Med Ausbild.

[33] Werwick K, Winkler-Stuck K, Hampe W, Albrecht P, Robra B-P (2015). Introduction of the HAM-Nat examination - applicants and students admitted to the medical faculty in 2012-2014. GMS Z Med Ausbild.

[34] Knorr M, Hissbach J (2014). Multiple mini-interviews: same concept, different approaches. Med Educ.

[35] Hissbach J, Sehner S, Harendza S, Hampe W (2014). Cutting costs of multiple mini-interviews - changes in reliability and efficiency of the Hamburg medical school admission test between two applications. BMC Med Educ..

[36] Zimmermann S, Klusmann D, Hampe W (2017). Correcting the predictive validity of a selection test for the effect of indirect range restriction. BMC Med Educ.

[37] Thorndike RL (1949). Personnel selection; test and measurement techniques.

[38] Pfaffel A, Schober B, Spiel CA (2016). Comparison of three approaches to correct for direct and indirect range restrictions: a simulation study. Pract Assess Res Eval.

[39] Lutz JG (1983). A method for constructing data which illustrate three types of suppressor variables. Educ Psychol Meas.

[40] Amelang M, Wottawa H, Einige Probleme d (1980). “Testfairness” und ihre Implikationen fuer Hochschulzulassungsverfahren. Diagnostica.

[41] Dunning D. Self-insight: Roadblocks and detours on the path to knowing thyself: Psychology Press; 2012.

[42] Urlings-Strop LC, Stegers-Jager KM, Stijnen T, Themmen APN (2013). Academic and non-academic selection criteria in predicting medical school performance. Medical Teacher.

[43] Urlings-Strop LC, Themmen APN, Stijnen T, Splinter TAW (2011). Selected medical students achieve better than lottery-admitted students during clerkships. Med Educ.

[44] Griffin B (2014). The ability to identify criteria: its relationship with social understanding, preparation, and impression Management in Affecting Predictor Performance in a high-stakes selection context. Hum Perform.

